# Association of more negative attitude towards commencing insulin with lower glycosylated hemoglobin (HbA1c) level: a survey on insulin-naïve type 2 diabetes mellitus Chinese patients

**DOI:** 10.1186/s40200-016-0223-0

**Published:** 2016-02-24

**Authors:** Sau Nga Fu, Carlos King Ho Wong, Weng Yee Chin, Wan Luk

**Affiliations:** 1 Department of Family Medicine and Primary Health Care, Kowloon West Cluster, Hospital Authority, G/F, Ha Kwai Chung General Outpatient Clinic, 77 Lai Cho Road, Kwai Chung, Kowloon, Hong Kong S.A.R., China; 2 Department of Family Medicine and Primary Care, Li Ka Shing Faculty of Medicine, University of Hong Kong, 3/F., 161 Main Street, Ap Lei Chau Clinic, Ap Lei Chau, Hong Kong S.A.R., China

**Keywords:** Questionnaires, Insulin, Attitude, Primary care, Fear

## Abstract

**Background:**

Delay in commencing insulin among type 2 Diabetes Mellitus (DM) patients is common.  One of the reasons is patients' psychological insulin resistance, which is particularly prevalent in Chinese patients. This study examined the correlation between socio-demographic and clinical characteristics; and attitudes towards commencing insulin in Chinese primary care patients.

**Method:**

A cross-sectional survey was conducted on 303 insulin-naïve Type 2 DM patients recruited from 15 primary care clinics across Hong Kong using the Chinese Attitudes to Starting Insulin Questionnaire (Ch-ASIQ). Subject selection criteria were patients on maximal oral anti-diabetes treatment who needed to commence insulin therapy. Linear regression was used to identify correlations between age, sex, educational level, occupation, body mass index, diabetes disease duration, laboratory test indicating disease control and biochemical markers including glycosylated hemoglobin (HbA1c) level, low density lipoprotein level and estimated glomeruli filtration rate, and presence of diabetic complications with the four sub-scales (self-image and stigmatization; factors promoting self-efficacy; fear of pain or needles; time and family support ) and the overall Ch-ASIQ score.

**Results:**

The most prevalent negative attitude was ‘fear of needle injections’ (70.1 %). The most common positive attitude was ‘I can manage the skill of injecting insulin’ (67.5 %). The mean Ch-ASIQ score of 2.50 (S.D. = 0.38) was equal to the mid-score, which signified an overall ambivalent attitude among the study population. Women scored significantly higher in the fear of pain or needles subscale (*p* = 0.011) and had an overall more negative attitude towards commencing insulin (*p* = 0.016). Subjects with lower HbA1c levels also had a significantly lower Ch-ASIQ sum score (*p* = 0.048) indicating a more negative attitude towards commencing insulin.

**Conclusion:**

In Chinese primary care patients with Type 2 DM, the need to commence insulin was associated with a number of negative emotions, which lead to a lower motivation to accept treatment. Perception of need as indicated by HbA1c level may be an important influencing factor determining a patient’s overall attitude towards starting insulin. Fortunately, in our setting, the injection technique does not appear to be a major barrier. However, needle fears are common, especially amongst women. Target interventions to acknowledge and help them to overcome their fears are essential before insulin treatment is commenced.

## Background

Current guidelines recommend that insulin treatment should be initiated as early as possible in patients with Type 2 diabetes mellitus (DM) who fail to achieve adequate glycemic control on oral drugs only, as early initiation of insulin has been shown to improve outcomes [1]. Delays in commencing insulin are commonly observed in studies conducted in the United States and the United Kingdom [2, 3], which have been shown to result in higher risks of DM complications [4]. Previous studies have identified that the reasons for delay are often related to psychological, cognitive and physical barriers from the perspectives of both patients and their physicians [5]. Patients’ reluctance to initiate insulin, termed ‘Psychological Insulin Resistance’ (PIR) [6] is an area of interest to researchers because health care professionals need to be informed on how to assist patients to overcome their barriers. There is evidence of ethnic differences in the reasons behind the resistance to starting insulin [7]. In Asia, and in particular Chinese patients with DM, a higher fear of injection and a greater perception of hardship towards using insulin was observed when compared with patients in Western settings [8, 9]. Women and patients with lower education levels have also been identified as having greater PIR [10].

Worldwide, as the prevalence of Type 2 DM increases, the number of patients needing to commence insulin is foreseeably going to rise [11]. Health care professionals will start to encounter more and more patients with PIR, and there will be a need for more evidence-based strategies on how to help patients overcoming their barriers to commencing insulin.

The predictors and factors associated with specific key attitudes towards insulin can inform the development of evidence-based interventions, to help patients to overcome their PIR and enhance acceptance to initiate insulin [12]. Therefore, this study aimed at exploring the relationship between socio-demographics and clinical characteristics (as indicated by their laboratory results and presence of diabetic complications), and attitudes towards commencing insulin in type 2 DM patients requiring insulin [13].

## Methods

### Participants

Subjects were recruited from 15 government-funded primary care general outpatient clinics (GOPCs) located in Kowloon, New Territories and Lantau Island of Hong Kong. Clinic nurses and doctors identified all eligible Chinese-speaking DM patients using the computerized laboratory result enquiry system and the drug dispensing system from July 2011 to March 2013. Inclusion criteria included: age between 18 and 80 years; on maximum recommended dosage of oral anti-diabetes treatment OADs (defined by either on Gliclazide 320 mg, Gliclazide modified release 120 mg, Glibenclamide 15 mg or metformin ≥2 g daily or Dipeptidyl peptidase-4 inhibitors or alpha glucosidase inhibitor); and the most recent HbA1c level ≥7.5 % (58.5 mmol/mol) within past 12 months indicating insufficient glycaemic control [1, 14]. Subjects who were pregnant, or unable to answer a questionnaire due to mental incapacity, or who had already commenced on insulin were excluded.

### Data collection

All eligible subjects were flagged in the computerized appointment system before their scheduled clinic appointment. Following the medical consultation with the GOPC doctor, the targeted subjects were approached by trained research assistants (RAs) who explained the study, obtained signed informed consent, and distributed the study questionnaire. Participants were encouraged to self-administer the traditional Chinese questionnaire as far as possible. However, as a large proportion of the patients attending the GOPCs are elderly and have poor literacy or eyesight, the RAs helped to administer the questionnaires using local Cantonese to those who were unable to complete the questionnaire by themselves.

#### Questionnaires

A locally validated instrument, the 13-item Chinese Attitudes to Starting Insulin Questionnaire (Ch-ASIQ) was used to assess patients’ attitudes to starting insulin. The psychometric properties of the Ch-ASIQ have been evaluated and found to have four sub-scales consisting of 3 items related to ‘Self-image and stigmatization’; 5 items related to ‘Factors promoting self-efficacy; 3 items related to ‘Fear of pain or needles’; and 2 items related to ‘Time and family support’ (Appendix) [13]. Responses to each item are scored using a 4-point Likert scale (Totally disagree = 1, Disagree = 2, Agree = 3; Totally Agree = 4). Individual scale scores are calculated by summing of the item responses. For two of the subscales ‘Factors promoting self-efficacy’ and ‘Time and family support’, higher scores indicates more positive attitudes towards the use of insulin. For the other two subscales ‘Self-image and stigmatization’ and ‘Fear of pain or needles’ a higher score indicates a more negative attitude towards insulin initiation. The weighted sum of all four scale scores, converting two positively coded scales to negatively coded scales, calculates the Ch-ASIQ overall score with higher scores indicating more negative attitude to starting insulin. All subscale and overall scores range from 1 to 4, with a mid-point of score 2.5. The Cronbach’s alpha coefficients for the four factors were above 0.60 (0.62–0.80). It is the first psychometric validated questionnaire on barriers to starting insulin for diabetic patients in the primary care setting [13].

#### DM complications assessment

All Type 2 DM patients who attend the GOPC undergo routine laboratory tests, retinal photo or ophthalmologist assessment and nurse-led diabetes complication screening clinics under the Risk Factor Assessment & Management Programme (RAMP) [15, 16] which is conducted once every 1–2 years as part of their usual chronic disease managed care. Plasma glycosylated hemoglobin (HbA1c) level, estimated glomerular filtration rate (eGFR; ml min^−1^1.73 m^−2^) by Modification of Diet in Renal Disease Formula, lipid profile (including low density lipoprotein LDL; mmol/L), urinary albumin-to-creatinine ratio (ACR; mg/mmol) were assessed in associated hospital or Department of Health Central Laboratories. Presence of foot ulcers and DM neuropathy (by using 10-g monofilament plus testing vibration perception threshold) were assessed by trained GOPC nurses. Presence of diabetic retinopathy diagnosis was confirmed either by registered optometrist, ophthalmologist or GOPC physicians. Presence of DM complications, physician diagnosis of hypertension, peripheral vascular disease, ischemic heart disease and stroke, all the biomedical and socio-demographic data were reviewed via the Hospital Authority’s computerized patient records.

### Ethics

The Research Ethics Committee of the Kowloon West Cluster, Hospital Authority of Hong Kong granted research ethics approval of the research protocol.

## Statistical analysis

From the power analysis with a sample size of 303 patients recruited in this study, a 99.8 % power was achieved to detect an R-square of 0.126 attributed to 13 independent variables using an F-test with a significance level of 0.05. Hence, the sample size retained the sufficiently high power for regression analysis.

Descriptive statistics were calculated with median and interquartile ranges (IQR) for continuous variables, and frequency and proportion for categorical variables. Proportions of patients giving choices of “Agree” or “Totally Agree” for each item of Ch-ASIQ were calculated. Mean and standard deviation of 13 individual item scores and 4 scale scores were shown. Linear regressions were performed to explore the associations of scale scores and overall score of Ch-ASIQ with patients’ socio-demographic and clinical characteristics. IBM SPSS Statistics for Windows, Version 21.0 statistical software was used to conduct descriptive and linear regression analyses.

## Results

Of the 306 potential subjects approached, three refused to participate (response rate =99.0 %). The socio-demographic and clinical characteristics of the subjects are shown in Table 1. Typical of the patient population attending government-funded primary care clinics, these subjects were elderly (median age 63, interquartile range (IQR) = 54–70), had lower levels of educational attainment (56.5 % primary or below), and only one third were in full-time employment.

**Table 1:** Sociodemographic and clinical charactistics of type 2 diabetic patients at baseline

Characteristics	Total (*N* = 303)
*Sociodemographic*	
Age (year; median, IQR)	63 (54–70)
Gender (%)	
Male	136 (44.9 %)
Female	167 (55.1 %)
Education (%)	
No formal education	44 (15.4 %)
Primary	117 (41.1 %)
Secondary	107 (37.5 %)
Tertiary	17 (6.0 %)
Occupation (%)	
Full time work	90 (32.8 %)
Unemployed/retired	82 (29.9 %)
Housewife	99 (36.1 %)
Part time	3 (1.1 %)
Mode of Administration (%)	
Self	104 (34.3 %)
Interviewer	199 (65.7 %)
*Clinical*	
Duration of DM (year; median, IQR)	11 (7–16)
Number of DM drug (%)	
On monotherapy	4 (1.3 %)
On two drug therapy	287 (94.7 %)
On three drug	12 (4.0 %)
BMI (kg/m^2^; median, IQR)	25.2 (22.6–27.7)
BMI cutoff	
Underweight (<18.5)	5 (1.7 %)
Normal range (≥18.5–<23)	80 (26.8 %)
Overweight (≥23–<25)	52 (17.4 %)
Obese (≥25)	162 (54.2 %)
HbA1c (%; median, IQR)	8.3 (7.9–9.1)
LDL (mmol/L; median, IQR)	2.5 (2.0–3.0)
eGFR (ml/min/1.73 m^2^ ; median, IQR)	88.0 (70.5–108.0)
History of	
Hypertension (%)	246 (81.2 %)
Nephropathy (%)	49 (16.8 %)
Footulcer (%)	4 (1.4 %)
Neuropathy (%)	6 (2.0 %)
PVD (%)	0 (0.0 %)
Retinopathy (%)	165 (57.5 %)
IHD (%)	5 (1.7 %)
Stroke (%)	14 (4.8 %)

*IQR* interquartile range, *BMI* body mass index, *LDL* low-density lipoprotein, *eGFR* epidermal growth factor receptor

The median duration of Type 2 DM was 11 years (IQR = 7 to 16 years) and median HbA1c level was 8.3 % (67.2 mmol/mol) [IQR = 7.9 % (62.8 mmol/mol) to 9.1 % (76.0 mmol/mol)] indicating overall very poor level of glycemic control. Most subjects (94.7 %) were on two kinds of OADs (combination of metformin with another sulphonylurea) while 1.3 % were on monotherapy and 4 % were on 3 kinds of OADs (combination of metformin with sulphonylurea and dipeptidyl peptidase-4 inhibitors or alpha glucosidase inhibitor). Seventy-two percent of them were either obese (54.2 %) or overweight (17.4 %). Most of them (81.2 %) had concomitant hypertension, while few subjects had coexisting stroke (4.8 %) and ischemic heart disease (1.7 %). Their LDL level (median = 2.5 mmol/L,IQR 2.0–3.0) and estimated glomerular filtration rate eGFR (median =88.0 ml/min/1.73 m^2^, IQR 70.5–108.0) were overall clinically optimal. The most common DM complication in this population was retinopathy (57.5 %), followed by nephropathy (16.8 %) and neuropathy (2.0 %).

Descriptive statistics of the 13 items and overall score of Ch-ASIQ are shown in Table 2. Due to missing values, 8 (2.6 %), 30 (9.9 %), 10 (3.3 %), 18 (5.9 %), and 41 (13.5 %) patients were excluded from the analysis of scale 1, scale 2, scale 3, scale 4 and the overall Ch-ASIQ score, respectively. Mean scores for each subscale were calculated. Higher mean subscale scores in scale 1 (self-image and stigmatization) and scale 3 (fear of pain or needles) showed more negative attitudes towards insulin, whilst higher mean subscale scores in scale 2 (factors promoting self-efficacy) and scale 4 (Time & family support) showed more positive attitudes. The overall Ch-ASIQ scored 2.50 (S.D. 0.38) which was the mid-point score. It indicated overall ambivalence to starting insulin. The highest three negative attitudes regarding starting insulin were “I am afraid of needle injection” (70.6 %), followed by “injecting insulin is painful” (66.9 %), and “There is no social support available if I have to inject insulin” (55.6 %). The highest three positive attitudes towards starting insulin all belonged to scale 2: the factors promoting self-efficacy: “I can manage the skill of injecting insulin” (67.5 %); followed by “I can pay as close attention to my diet as my insulin treatment required” (66.7 %); and “insulin can help control blood glucose and prevent complications” (63.7 %).

**Table 2:** Mean scores of Ch-ASIQ overall, each individual item and scale, and distribution of responses to individual items

Scale/individual item			Mean ± SD	Agree/Totally agree (%)
Scale 1^a^	Self image and stigmatization (*n* = 295)		2.44 ± 0.69	
	Item #1	I worry that people will know I have diabetes if I am on insulin treatment	2.36 ± 0.85	121 (40.33 %)
	Item #2	Injecting insulin is embarrassing, I worry about being seen when I inject insulin	2.49 ± 0.81	147 (49.16 %)
	Item #3	If I have to inject insulin, it makes me feel like a drug addict	2.45 ± 0.80	133 (44.93 %)
Scale 2^b^	Factors promoting self-efficacy (*n* = 273)		2.60 ± 0.47	
	Item #4	I have up-to date knowledge about diabetes management	2.60 ± 0.83	189 (63.21 %)
	Item #5	Insulin can help control blood glucose and prevent complications	2.65 ± 0.68	181 (63.73 %)
	Item #6	I can manage the skill of injecting insulin	2.72 ± 0.75	201 (67.45 %)
	Item #7	There is social support available if I have to inject insulin	2.37 ± 0.68	131 (44.41 %)
	Item #8	I can pay as close attention to my diet as my insulin treatment requires. For example, I may need to take a snack or reduce my eating amount appropriately	2.67 ± 0.66	196 (66.67 %)
Scale 3^a^	Fear of pain or needles (*n* = 293)		2.76 ± 0.62	
	Item #9	Injecting insulin is painful	2.79 ± 0.73	200 (66.89 %)
	Item #10	I am afraid of needle injections	2.91 ± 0.86	211 (70.57 %)
	Item #11	I worry about needing to perform home blood sugar monitoring	2.59 ± 0.82	158 (53.02 %)
Scale 4^b^	Time & family support (n = 285)		2.51 ± 0.62	
	Item #12	I can spare enough time to perform insulin injections	2.58 ± 0.71	176 (59.46 %)
	Item #13	My family will support me to inject insulin	2.45 ± 0.73	133 (45.70 %)
Ch-ASIQ Overall^a^ (*n* = 262)			2.50 ± 0.38	

Note:

^**a**^Higher scores indicate the greater level of barriers to insulin

^**b**^Higher scores indicate the lower level of barriers to insulin

Results of the linear regression analysis showing the factors and the scores of Ch-ASIQ are shown in Table 3. More negative attitudes to starting insulin were found in women (*p* = 0.01 6). Being female accounted for 16.4 % of variance of the negative attitude towards insulin. While the most significant factors among women was fear of pain or needles (*p* = 0.011), which accounted for 26.2 % of the variance of the negative attitude. Interviewer administered subjects had lower self-efficacy when compared with self-administered subjects. Subjects with lower HbA1c levels had significantly higher barriers to starting insulin. Subjects on larger number of OADs had more factors promoting self-efficacy for insulin therapy (*p* = 0.04) including: better up-to-date knowledge (item#4);better social support for insulin treatment (item#7); higherbelief that insulin can help control blood sugar (item#5); better confidence that they can manage the skill of injecting insulin (item#6) and diet control (item#8). Age, education level, working status, duration of DM, coexisting hypertension and complicated with retinopathy showed no significant differences in the overall scores and subscales of Ch-ASIQ.

**Table 3:** Factors associated with the Ch-ASIQ scale and overall scores

		Scale 1^a^ self image and stigmatization		Scale 2^b^ factors promoting self-efficacy		Scale 3^a^ fear of pain or needles		Scale 4^b^ time & family support		Ch-ASIQ overall^a^	
	Factor	Coeff (95 % CI)	P-value^c^	Coeff (95 % CI)	P-value^c^	Coeff (95 % CI)	P-value^c^	Coeff (95 % CI)	P-value^c^	Coeff (95 % CI)	P-value^c^
*Sociodemographic*											
Age	Age	−0.004 (−0.016,0.008)	0.516	−0.008 (−0.016,0.001)	0.066	−0.003 (−0.014,0.008)	0.566	0.000 (−0.012,0.011)	0.945	0.003 (−0.005,0.010)	0.484
[Sex = 2]	Female	0.163 (−0.056,0.381)	0.145	−0.100 (−0.256,0.055)	0.206	0.260 (0.060,0.461)	**0.011**	−0.052 (−0.261,0.157)	0.626	0.164 (0.031,0.297)	**0.016**
[Education_r = 2]	Education (No formal education)		0.516		0.279		0.845		0.496		0.893
[Education_r = 1]	• Secondary or above	0.146 (−0.139,0.432)	0.314	0.162 (−0.041,0.365)	0.117	0.059 (−0.199,0.317)	0.654	0.148 (−0.123,0.418)	0.285	−0.039 (−0.212,0.134)	0.656
[Education_r = 0]	• Primary	0.053 (−0.214,0.320)	0.696	0.096 (−0.094,0.285)	0.322	0.071 (−0.169,0.312)	0.562	0.146 (−0.106,0.398)	0.256	−0.037 (−0.199,0.125)	0.655
[Occupation_r = 3]	Working Status (Working)		0.341		0.969		0.504		0.274		0.646
[Occupation_r = 2]	• Housewife	−0.152 (−0.414,0.110)	0.257	0.024 (−0.161,0.208)	0.802	0.135 (−0.106,0.376)	0.272	0.149 (−0.104,0.402)	0.249	−0.045 (−0.205,0.115)	0.583
[Occupation_r = 1]	• Unemployed/retired	−0.170 (−0.416,0.076)	0.175	0.008 (−0.164,0.180)	0.925	0.100 (−0.123,0.324)	0.379	0.187 (−0.051,0.426)	0.123	−0.070 (−0.217,0.078)	0.354
	• Interviewer Administration	0.074 (−0.109,0.258)	0.427	−0.167 (−0.294, −0.040)	**0.010**	0.140 (−0.029,0.309)	0.105	0.063 (−0.113,0.238)	0.484	0.104 (−0.005,0.212)	0.060
*Clinical*											
DM_Duration	Duration of DM	−0.012 (−0.028,0.003)	0.120	0.005 (−0.005,0.016)	0.329	0.002 (−0.012,0.016)	0.761	0.004 (−0.011,0.019)	0.594	−0.006 (−0.015,0.003)	0.208
Drug_no	Number of DM drug	−0.138 (−0.489,0.214)	0.443	0.256 (0.011,0.500)	**0.040**	−0.055 (−0.373,0.264)	0.735	0.003 (−0.326,0.332)	0.985	−0.166 (−0.371,0.038)	0.111
[BMI2_r = 3.00]	BMI (Underweight/Normal)		0.390		0.225		0.434		0.181		0.906
[BMI2_r = 2.00]	• Obese	−0.143 (−0.351,0.065)	0.178	−0.128 (−0.275,0.018)	0.086	−0.125 (−0.314,0.065)	0.197	0.135 (−0.065,0.335)	0.186	−0.016 (−0.141,0.108)	0.800
[BMI2_r = 1.00]	• Overweight	−0.121 (−0.395,0.153)	0.386	−0.094 (−0.285,0.097)	0.334	−0.072 (−0.328,0.183)	0.578	0.237 (−0.025,0.499)	0.076	−0.037 (−0.199,0.126)	0.659
HbA1c	HbA1c (Unit in %)	−0.034 (−0.110,0.042)	0.385	0.049 (−0.004,0.102)	0.071	−0.041 (−0.113,0.030)	0.255	0.037 (−0.037,0.111)	0.325	−0.046 (−0.091,0.000)	**0.048**
LDL	LDL (Unit in mmol/L)	−0.064 (−0.178,0.050)	0.271	0.024 (−0.053,0.101)	0.540	−0.043 (−0.148,0.061)	0.419	0.050 (−0.057,0.157)	0.358	−0.057 (−0.123,0.009)	0.092
eGFR	eGFR (Unit in ml/min/1.73 m^2^)	0.002 (−0.002,0.006)	0.448	0.002 (−0.001,0.005)	0.186	0.000 (−0.003,0.004)	0.828	0.001 (−0.002,0.005)	0.443	−0.001 (−0.003,0.002)	0.662
History of											
[HT = 1]	• Hypertension	0.131 (−0.088,0.349)	0.242	0.089 (−0.066,0.243)	0.261	0.004 (−0.199,0.207)	0.971	−0.006 (−0.215,0.203)	0.955	−0.022 (−0.155,0.112)	0.751
[Nephropathy = 1]	• Nephropathy	0.250 (−0.025,0.524)	0.074	0.055 (−0.133,0.242)	0.569	0.292 (0.045,0.538)	**0.020**	−0.060 (−0.317,0.197)	0.649	0.109 (−0.048,0.266)	0.175
[Retinopathy = 1]	• Retinopathy	0.059 (−0.115,0.233)	0.509	0.055 (−0.066,0.176)	0.376	0.079 (−0.080,0.239)	0.328	0.031 (−0.134,0.196)	0.712	0.013 (−0.090,0.115)	0.810

Note:

^a^Higher scores indicate the greater level of barriers to insulin

^b^Higher scores indicate the lower level of barriers to insulin

^c^Bold indicates the statistically significant with P-value <0.05

## Discussion

The study population characteristics showed that they were representative of the type 2 DM patients attending GOPCs in Hong Kong [17]. Despite their overall older age 63 (IQR: 54–70) and low education level (56.5 % attained primary education or below), more than 65 % of them believed that they could manage theinjection skill and diet modifications associated with insulin treatment (item #6 & #8) (Appendix), which had been found to be a barrier to insulin in previous studies [18]. These positive attitudes could be acknowledged and reinforced when health care professionals tried to introduce insulin to their patients. On the other hand, a negative self-image and fear on insulin injection might cause much emotional distress, which could potentially outweigh their perceived confidence in managing the treatment. Understanding such commonly encountered ambivalent negative emotions and positive cognitions [5] is an essential basic step to build trusting relationships with patients. Training in proper injection techniques and provision of a structured program of face-to-face and telephone support are helpful strategies in initiating insulin in the primary are setting [12]. Involvement of a multidisciplinary health care team, provision of equipment for injection, and home sugar monitoring appear to be essential elements for the successful initiation of insulin in primary care setting [19].

As with many other studies, fear of pain and needles was an important barrier to starting insulin [9, 10] especially in women [8, 20]. Patients with a needle phobia would typically want to avoid medical treatments involving needles [20]. This finding was consistent to patients’ avoidance of insulin injection despite their poor glycemic control. Health care professionals can consider different interventions to overcome needle phobia, such as use of different injection devices or injection sites,[19, 21] and specific counselling to overcome needle phobias [22].

Subjects with lower HbA1c were observed to have greater attitudinal barriers to starting insulin. This seems to be unique to our setting as no such association has been reported in previous studies [8, 10, 23]. Patients may understand their glycemic control as “better” or “acceptable” evidenced by lower HbA1c level. This finding could possibly be explained by previous study finding that the refusal of insulin was caused by patients did not see the necessity for insulin and they actively seek ways to control blood sugar within insulin [5]. Patients may perceive insulin as the last resorts [24]. Subjects in this study had recent HbA1c levels >7.5 %, which signified poor control yet, they may not have perceived that their DM control was sufficiently poor to require insulin. Such disagreements between patient perceptions of glycemic control with the actual HbA1c level may need to be addressed during patient education process. Patient empowerment programs involving self-awareness of treatment target and control level may be helpful to define the best time to starting insulin [25, 26].

Subjects using more types of OADs were found to have significantly higher scores in the subscale related to promoting self-efficacy, which was a positive factor for starting insulin. Adding more types of OADs may be one of the acceptable ways to control blood sugar without insulin. Yet, more types of OADs will raise the patients’ awareness of poor glycemic control. They may possibly be better mentally prepared to accept insulin therapy. Health care professionals can target the patients on multiple OADs for insulin education program.

There were several limitations in this study. The questionnaires were interviewer-administered in majority of the subjects as our patient population has poor literacy levels. It is possible that the Ch-ASIQ score may differ if self-administered. The observed correlation might not be applicable to other cultural or age group patients because all subjects were Chinese in primary care outpatient clinics and most of them were older adults. The resulting score were asked attitude to insulin initiation by cross sectional survey, which might not reflect actual patient refusal behavior when physicians prescribed insulin to them.

## Conclusion

In summary, the most significant barriers to commencement of insulin in poorly controlled Type 2 DM Chinese primary care patients appears to be fear of pain and needles. However, most patients are aware of the clinical effectiveness of insulin and have sufficient confidence to learn the skill of insulin injection and how to manage their diet when taking insulin. Women and subjects with lower HbA1c had more negative attitude towards starting insulin. Women had higher fear of pain or needles. Subjects on more types of OADs may be more ready to receive new DM treatment information. To better address conflicting attitudes towards insulin, health care professionals can offer tailored education, which has been shown to be beneficial to poorly controlled DM patients to specifically address the negative attitudes identified in this study.

## Appendix

**Table 4:** Chinese Attitudes to Starting Insulin Questionnaire (Ch-ASIQ)

Item	English version	Totally disagree	Disagree	Agree	Totally agree
(a) Self-image and stigmatization					
1	I worry that people will know I have diabetes if I am on insulin treatment	o	o	o	o
2	Injecting insulin is embarrassing, I worry about being seen when I inject insulin	o	o	o	o
3	If I have to inject insulin, it makes me feel like a drug addict	o	o	o	o
(b) Factors promoting self-efficacy					
4	I have up-to date knowledge about diabetes management	o	o	o	o
5	Insulin can help control blood glucose and prevent complications	o	o	o	o
6	I can manage the skill of injecting insulin	o	o	o	o
7	There is social support available if I have to inject insulin	o	o	o	o
8	I can pay as close attention to my diet as my insulin treatment requires. For example, I may need to take a snack or reduce my eating amount appropriately	o	o	o	o
(c) Fear of pain or needles					
9	Injecting insulin is painful	o	o	o	o
10	I am afraid of needle injections	o	o	o	o
11	I worry about needing to perform home blood sugar monitoring	o	o	o	o
(d) Time & Family Support					
12	I can spare enough time to perform insulin injections	o	o	o	o
13	My family will support me to inject insulin	o	o	o	o

## Abbreviations

ACR: Albumin-to-creatinine ratio
Ch-ASIQ: Chinese attitudes to starting insulin questionnaire
DM: Diabetes mellitus
eGFR: Glomerular filtration rate
GOPCs: General outpatient clinics
HbA1c: Glycosylated hemoglobin
IQR: Interquartile ranges
LDL: low density lipoprotein
OADs: Oral anti-diabetes treatment
PIR: Psychological insulin resistance
RAMP: Risk factor assessment & management programme
RAs: Research assistants
